# Dietary Protein and Amino Acid Intake: Links to the Maintenance of Cognitive Health

**DOI:** 10.3390/nu11061315

**Published:** 2019-06-12

**Authors:** Jordan M. Glenn, Erica N. Madero, Nick T. Bott

**Affiliations:** 1 Exercise Science Research Center, Department of Health, Human Performance and Recreation, University of Arkansas, Fayetteville, AR 72701, USA; 2 Neurotrack Technologies, 399 Bradford St, Redwood City, CA 94063, USA; erica@neurotrack.com (E.N.M.); nick@neurotrack.com (N.T.B.); 3 Clinical Excellence Research Center, Department of Medicine, School of Medicine, Stanford University, Stanford, CA 94305, USA

**Keywords:** protein, amino acid, Alzheimer’s disease, dementia, cognition, cognitive decline, risk factors

## Abstract

With the rapid growth in the aging population, there has been a subsequent increase in the rates of Alzheimer’s disease and related dementias (ADRD). To combat these increases in ADRD, scientists and clinicians have begun to place an increased emphasis on preventative methods to ameliorate disease rates, with a primary focus area on dietary intake. Protein/amino acid intake is a burgeoning area of research as it relates to the prevention of ADRD, and consumption is directly related to a number of disease-related risk factors as such low-muscle mass, sleep, stress, depression, and anxiety. As a result, the role that protein/amino acid intake plays in affecting modifiable risk factors for cognitive decline has provided a robust area for scientific exploration; however, this research is still speculative and specific mechanisms have to be proven. The purpose of this review is to describe the current understanding of protein and amino acids and the preventative roles they play with regard to ADRD, while providing future recommendations for this body of research. Additionally, we will discuss the current recommendations for protein intake and how much protein older adults should consume in order to properly manage their long-term risk for cognitive decline.

## 1. Introduction

Characterized by declines in mental ability severe enough to interfere with daily life, Alzheimer’s disease and related dementias (ADRD) pose serious worldwide challenges as they relate to patients, their caregivers, and health care systems. Projections estimate the global prevalence of ADRD to triple to greater than 130 million individuals between 2015 and 2050 [1]. ADRD is considered one of the world’s most expensive health conditions, with the lifetime cost of care for diagnosed individuals estimated at US$321,000/person [2]. In the United States alone, ADRD costs are projected to grow from $186 billion in 2018 to $750 billion in 2050 (400% increase), while dementia diagnoses are projected to increase by ~150%, from 5.5 million to 13.8 million in that same timespan [2]. Furthermore, caregivers provided an estimated 18.4 billion hours of care (valued at over $232 billion) to diagnosed individuals [2].

Although there is a massive cost associated with the disease once diagnosed, ADRD tends to go undetected for long periods of time due to a prolonged preclinical phase [3,4]. In this phase, neuronal and neurobiological changes can occur for years or decades before noticeable symptoms appear. Prior to the development of clinically detectable cognitive issues, people commonly experience a phenomenon termed subjective cognitive decline (SCD) [5]. SCD is defined as subjective changes in memory and cognition that are perceived by the individual, but are not associated with clinically measurable abnormalities [3,6]. Individuals experiencing SCD are considered at risk for developing dementia, specifically AD [7,8,9]. If left unchecked, SCD can evolve into mild cognitive impairment (MCI), an intermediate between normal cognitive function and diagnosable dementia [6,10]. Given the aforementioned cost burden associated with ADRD and the current lack of effective pharmaceutical agents, there is a need to develop non-pharmacological interventions that can slow or delay pathological cognitive decline.

**Figure 1:**
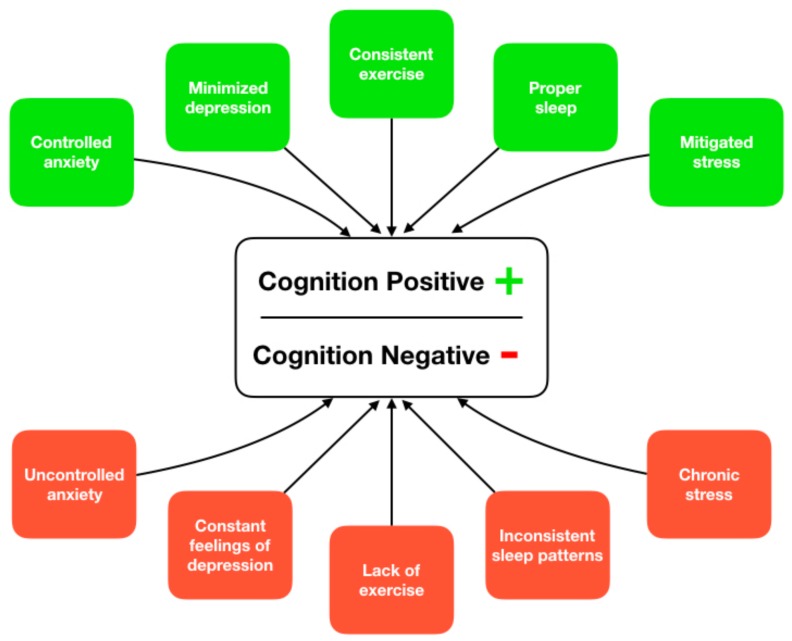
Supported paths to ameliorate or increase risk of cognitive decline through lifestyle-based activities.

It is increasingly accepted that the causes of ADRD are multifactorial and a combination of risk factors (modifiable and unmodifiable) are implicated in disease onset and progression [11]. As a result, it is difficult to categorize the treatment of ADRD as a single factor solution and it is instead recommended that researchers take a multifactorial approach, focusing on behavioral risk factors associated with disease development and progression [12]. Some of the primary modifiable risk factors associated with ADRD are exercise, sleep, stress, depression, and anxiety; and improvement in any of these factors may reduce overall risk for disease progression (Figure 1). Furthermore, the ability to improve these risk factors at an individual level can have an overall positive effect on cognitive decline trajectories.

One of the primary preventive measures recommended to ameliorate cognitive decline is diet and multiple dietary factors have already been investigated [13,14,15]. An in-depth explanation of diet and cognitive decline can be found elsewhere [16] and is beyond the scope of this review. Of the three major macronutrients, protein, and its constituent amino acids, are indispensable components of dietary patterns, essential for maintaining cellular function and integrity (including brain cells). In fact, a large-scale investigation totaling almost 1000 subjects found that a diet high in protein intake was associated with a reduced overall risk of MCI or ADRD [17]. The researchers suggested that these results may be attributed to the fact that low amino acid consumption is associated with decreased intake of essential proteins required for neurotransmitter synthesis (e.g., tryptophan can cross the blood–brain barrier and is a serotonin precursor). Studies utilizing animal models suggest that tryptophan transport across the blood–brain barrier may decrease with ageing [18] and, should this hold true in humans, reduced intake of proteins in the elderly may adversely impact neuronal function.

Insufficient protein intake is associated with decrements in modifiable risk factors for ADRD such as exercise [19], sleep [20], stress, depression and anxiety [21,22,23]. Socioeconomic status (SES) also tends to affect protein intake with lower SES leading to decreased rates on consumption [24,25]. Approximately 10–25% of older adults consume less protein than the Recommended Dietary Allowance (RDA) and 5–9% of older adults consume less than the Estimated Average Requirement (EAR) of 0.66g/kg·day [26]. Important to note is that the EAR is the mean intake at which the needs of the healthy population are met, which means that this intake level is insufficient for approximately half of the population [26]. As a result, the percentage of older adults at risk for inadequate protein intake is potentially larger, and even more so for older patients with chronic or acute illnesses.

**Figure 2:**
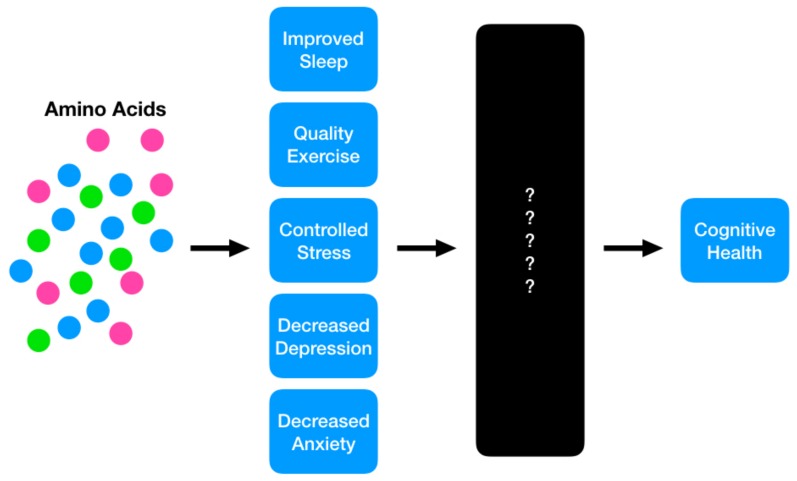
The proposed model in which protein and its constituent amino acids may play a role in mitigating risk for future cognitive decline. The current state of the science suggests a strong potential for these nutritional interventions to achieve positive benefits; however, the causal mechanisms are still a ‘black box,’ requiring future investigation.

The role that protein and its constituent amino acids play in ameliorating modifiable risk factors for cognitive decline, combined with the fact that a significant proportion of older adults do not consume adequate levels of the macronutrient, have made this an intriguing area for scientific exploration. However, the causal nature of this relationship is poorly understood due to the lack of rigorous studies on amino acids and cognitive decline (Figure 2) and as a result, many mechanisms remain speculative. Nevertheless, a number of studies have uncovered correlations that begin to demonstrate the role that amino acids play in cognitive health. After summarizing the current understanding of protein and amino acids with regard to known ADRD risk factors, this review will provide future recommendations for this body of research, in an effort to support a transition from associational work to more robust scientific explorations that further elucidate the underlying mechanisms of action.

## 2. Methods

All empirical articles included in this review were published in peer-reviewed journals. No restriction was placed on the year, language, study setting, or country in which the article was originally published. A study was included in the review if it was peer-reviewed and had multiple subjects (no case reports). Google and PubMed searches were performed to identify additional articles that may have been missed in the initial database searches. A Boolean search strategy was conducted with the following keywords and logic: (“exercise” OR “physical activity” OR “fitness” OR “health” OR “sleep” OR “stress” OR “anxiety” OR “depression” AND (“protein” OR “amino acids”) AND (“Alzheimer’s disease” OR “dementia” OR “cognitive impairment” OR “cognitive decline”) and all their derivatives as well as further investigation into the bibliographies of relevant papers. The review of literature is divided into the following sections: (a) exercise, (b) sleep, (c) mental health (stress, depression, anxiety), (d) protein recommendations/guidelines for older adults.

## 3. Exercise

Exercise and dietary protein/amino acid intake are classically linked, with this evidence spanning a gamut of areas including, but not limited to, exercise performance [27], frailty [28], and age-related chronic diseases (i.e., sarcopenia) [29]. Additionally, there is a vast body of literature suggesting that exercise itself has a direct impact on long-term cognition. To date, however, there is little evidence bridging the gap between these two entities. This section will depict this potential link, as well as describe the current, albeit limited, body of literature available on amino acid intakes and cognitive decline.

In order to understand the impact that exogenous protein intake may have on cognition, it is first important to delineate the independent relationship between muscle mass and cognitive health. Important work in this area has been conducted by Burns et al. [30], who evaluated healthy individuals and those diagnosed with early-stage AD. All subjects were evaluated on measures of dual-energy x-ray absorptiometry (DXA), brain magnetic resonance imaging (MRI), and neuropsychological testing. When compared with healthy controls, individuals diagnosed with early-stage ADRD demonstrated decreased levels of muscle mass as measured through DXA; these differences persisted even after controlling for sex. The study also found that decreases in the volume of white matter in the brain, whole brain volume, and lower cognitive performance were all significantly associated with reduced lean mass [30]. Importantly, total body fat and body fat percentage were not significantly different between individuals with and without dementia, nor were they associated with cognitive ability or brain volume. These results indicate that lean mass, as opposed to more classic body composition measures (i.e., body mass index/body fat), may be more sensitively associated with dementia risk. While the causal link between lean mass and cognitive decline cannot be directly inferred from this investigation, it is proposed that physical activity may attenuate the structural and functional brain and body changes associated with ADRD and aging [31]. Therefore, a lack of such activity, leading to decreases in muscle mass, may result in an accelerated decline in cognitive function.

In addition to lean muscle mass, muscular strength should also be considered with regard to cognitive decline. This concept was explored through a longitudinal investigation where muscular strength was individually measured from nine muscle groups in over 900 community-dwelling older adults without a baseline diagnosis of ADRD [32]. Muscle groups were evaluated individually and summarized into a composite measure. After approximately 4 years, results showed that each 1 unit increase in baseline muscle strength was associated with an approximately 43% decrease in the risk of developing ADRD. This association remained after controlling for factors such as body mass index, physical activity, pulmonary function, vascular risk factors, vascular diseases and apolipoprotein E4 status. Furthermore, increased muscle strength was associated with a slower rate of decline in global cognitive function and a decreased risk of MCI [32]. Combined with the previous investigation, this provides further evidence that exercise may be a protective mechanism for maintaining cognitive health.

Given that low muscle mass and muscular strength are significant risk factors for cognitive decline, preservation of both becomes increasingly important with advancing age. From a physical perspective, protein intake helps preserve muscle mass [33,34]. However, a multitude of age-related factors contribute to reduced protein consumption including decreased hunger, diminished oral health, and/or sensory loss of taste and smell (Table 1).

When combined, these factors tend to result in a negative protein turnover rate, ultimately leading to skeletal muscle catabolism or breakdown [35]. In skeletal muscle, protein turnover rate comprises the net result of muscle protein synthesis and protein breakdown [36,37] (see Tipton and Wolfe [38] for an in-depth description of the mechanistic process between protein metabolism and muscle growth). As a result, increased protein intake keeps this equation net positive, ultimately preserving or potentially increasing muscle mass.

Conversely, a continual protein turnover rate that is net negative, due to a consistent lack of adequate protein consumption or related factors, can lead to muscle wasting and eventually disease states such as sarcopenia. Sarcopenia is directly characterized by marked muscle atrophy, dynapenia, and reduced physical function, all of which are associated with decreased cognition [35,39,40]. Previous literature has indicated 12.5% of individuals with MCI and 23.3% of individuals with ADRD are comorbid with sarcopenia [41], suggesting that alleviating sarcopenic risk may also help reduce the risk of associated cognitive impairments.

Combining adequate amino acid intakes with exercise has been shown to improve lean mass in individuals clinically diagnosed with sarcopenia [41]. This study followed 150 sarcopenic women during a 3-month exercise protocol that included 30 min of strengthening exercises and 20 min of balance and gait training. After completion of the intervention, results demonstrated that the combination of exercise and amino acid supplementation was effective in enhancing not only muscle strength, but also muscle mass and walking speed in sarcopenic women [42]. However, it is important to note that the effectiveness of amino acid supplementation on muscle mass and muscular strength is not limited to individuals diagnosed with sarcopenia.

Another investigation evaluated the impact of protein supplementation on muscle mass, strength, and physical performance during prolonged resistance-type exercise training in frail elderly men and women [43]. The resistance-type exercise program included 2 sessions per week over a 24-week period; during this time, subjects were supplemented twice each day with either 15 g protein (30 g total/day) or a placebo. At the end of the intervention, lean body mass in the protein group increased from 47.2 kg to 48.5 kg while the placebo group experienced no changes. The between-group differences were isolated to lean mass; strength and physical performance improved significantly in both groups independent of dietary protein supplementation [43].

In all, these studies suggest a strong potential for the use of dietary protein/amino acids to enhance cognition through the participation in proper exercise programs designed to enhance muscular mass and strength. However, literature directly bridging the gap between amino acid intake and cognitive decline is sparse and further research needs to be conducted in order to properly understand the mechanisms behind this relationship. It is recommended that future studies investigate the longitudinal effects of adequate protein/amino acid intake when combined with properly developed exercise programs in baseline healthy populations. This design would allow researchers to determine the efficacy of this intervention by measuring adherence against conversion rates of MCI or ADRD.

## 4. Sleep

Sleep has not only been directly linked to short-term cognitive issues [44,45], but also the long-term advancement of ADRD [46,47,48,49]. Data from epidemiological investigations suggest that up to 45% of patients with ADRD report sleep disturbances [50,51] and these disturbances are directly correlated with advanced cognitive decline [50,52]. It is clear that these symptoms appear early on in the development of ADRD, with recent evidence linking even a single night of sleep deprivation with increased β-Amyloid accumulation in the brain [53]. For a mechanistic description of the relationship between sleep and the development of β-Amyloid, see Ju et al. [54] (a general review on the development of β-Amyloid in relation to ADRD can be found at Murphy and LeVine [55]). Furthermore, a link between sleep characteristics and cognitive decline in the elderly has been suggested, emphasizing the fact that sleep and cognition are closely related [53,56,57,58]. Specifically, daytime napping, night-time sleep duration, and excessive daytime sleepiness have been documented as clinical indicators of future cognitive decline in aging adults [58].

In general, when it comes to improving poor sleep quality, a multitude of nonpharmacological treatment options have been proposed including structured bedtime routines, decreased time in bed during the daytime hours, increased sunlight exposure, decreased nighttime noise/light, increased physical activity, and improved nutrition [20,50,59,60]. While the nutritional link to sleep quality was initially investigated in college-aged students [61,62], it has since been expanded to athletes [60], children [63], those at risk for chronic diseases (e.g., obesity) [64], and older adults [65] with positive results. Additionally, from this nutritional perspective, there is a growing body of evidence indicating that protein and/or amino acid intake can positively influence sleep quality and duration.

Neurotransmitters such as 5-hydroxytryptamine (5-HT), gamma-aminobutyric acid (GABA), orexin, melanin-concentrating hormone, acetylcholine, galanin, noradrenaline, and histamine have all been associated with the sleep-wake cycle. As a result, nutritional interventions that modulate the release and/or suppression of these neurotransmitters may have downstream effects on sleep. l-tryptophan is an amino acid that acts as an upstream precursor to both 5-HT and melatonin [65,66,67,68]. Melatonin is a hormone well documented to regulate sleep in humans via influence on the sleep–wake cycle. As such, nutritional interventions aimed at increasing melatonin through the manipulation of l-tryptophan are becoming prominent. l-tryptophan competes with other large neutral amino acids to cross the blood–brain barrier. Amino acid supplements containing high levels of l-tryptophan can increase the flow of l-tryptophan into the brain, and ultimately may play a significant role in promoting sleep quality [60].

Outside of l-tryptophan, there are other individual amino acids that may play a beneficial role in enhancing sleep. One of these amino acids is glycine, a non-essential amino acid typically created through de novo synthesis in humans, although small amounts (3–5 g) are typically consumed daily through the diet [69]. The effects of glycine supplementation were investigated in a double-blind, cross-over investigation including females experiencing subjective sleep problems [70]. When subjects consumed 3 g/day of glycine for 5 days, they experienced significant improvements in total scores on the Saint Mary’s Hospital Sleep Questionnaire (examines sleep over the preceding 24 h) and the Space-Aeromedicine Fatigue Checklist (examines feelings upon awakening) [70].

Another investigation using a similar 3 g dose found that glycine ingestion before bed improved subjective sleep quality and sleep efficacy (sleep time/in-bed time), lessened daytime sleepiness, and even improved memory recognition performance [71]. Additionally, polysomnography latency with regard to sleep onset (latency to the first appearance of stage 2 sleep) and slow wave sleep (latency to the first appearance of stage 3) were shortened with glycine ingestion. It should be noted that neither of the previous investigations evaluating glycine reported any adverse effects and this was further confirmed in a high dose study reporting that ingestion of 9 g/day, a three-fold increase from the previous studies, resulted in no self-reported adverse effects (with the exception of minor digestive issues), nor were there any carryover effects resulting in increased daytime sleepiness [72].

l-ornithine is another non-essential amino acid that may play a role in improving sleep; however, only a single investigation to date has studied its efficacy. When 52 healthy Japanese adults were supplemented with 400 mg/day of l-ornithine, self-reported insomnia was improved when compared to placebo-supplemented controls. For the intervention group, significant improvements were also observed for initiation and maintenance of sleep as well as sleep length [73]. While these results are positive, additional research is needed to substantiate these claims.

Further building on the thesis that protein/amino acids may be utilized for enhancing sleep, large-scale studies have been conducted investigating associations between macronutrient consumption and self-reported sleep duration. A study from the NHANES database including over 5000 subjects investigated the relationship between sleep duration with physical and mental health [74]. Researchers determined that individuals categorized as very short sleepers (<5 h/night) reported lower levels of protein consumption compared to normal sleepers (7–8 h/night) [74]. These results are similar to those reported by Kant et al. [75] and Haghighatdoost et al. [76], where results suggested that individuals with abnormal sleep cycles consumed significantly less protein when compared to those who maintained normal sleep cycles of 6–8 h/night.

It should be noted that these results are not universally supported. Another investigation looking at men and women aged 60–80 years determined that there was an inverse correlation between protein consumption and sleep duration, with decreased protein intake being related to greater sleep duration [65]. However, the authors from this investigation indicated that the subjects self-reported implausibly low caloric intakes, suggesting overall a significant underreporting of dietary intakes (not uncommon in self-report dietary designs) [77].

Taken together, these studies indicate that abnormal sleepers tend to consume diets lower in protein concentration and that there is a positive relationship between higher amounts of protein/amino acid intake and sleep duration. However, as reported by Santana et al. [65], these results are not ubiquitous, albeit controversial, and future randomized controlled studies must be conducted in order to further elucidate the relationship (see Table 2, adapted from Dashti et al. [20], for a list of study design and data analysis considerations).

As previously mentioned, sleep is a significant risk factor for ADRD. Additionally, protein and the supplementation of its constituent amino acids has been shown to potentially improve sleep quality and duration in a variety of populations. This clearly indicates the potentiality of a strong link between protein/amino acid intake and the improvement of sleep to ameliorate the progression of ADRD. However, the causal thesis of these relationships is yet unsubstantiated and more in-depth research is needed to concretize this hypothesis.

## 5. Mental Health

According to the World Health Organization, mental health is “a state of well-being in which the individual realizes his or her own abilities, can cope with the normal stresses of life, can work productively and fruitfully, and is able to make a contribution to his or her community [68].” When it comes to AD/dementia, there are various components of mental health that are associated with ADRD risk; a few of which include stress [78], depression [79], and anxiety [80,81]. All three of these components are suggested to be positively affected by certain protein/amino acid interventions which as a result, may play a role in mitigating cognitive decline.

Commonly defined as how the brain and body respond to any demand [82], stress can be triggered by any length (short/long), type (negative/positive), or reality (real/perceived) of change. When stress is prolonged over time, it is referred to as chronic stress and can have serious, detrimental effects on long-term physical and mental health. Over time, chronic stress can lead to a multitude of health-related concerns, including depression [83], anxiety [84] (both of which are risk factors for ADRD), and eventually cognitive decline [78]. Many studies have analyzed the mechanistic relationship between stress and cognitive decline in animal models [78,85,86,87,88], demonstrating that chronic stressors early in life tend to result in predisposition to enhanced AD-related pathogenesis. These results also seem to apply to humans, as demonstrated by the Rush Memory and Aging Project. In this project, investigators tested patients for features of neuroticism and found that patients that scored high for “distress proneness” were 2.7-fold more likely to be diagnosed with ADRD in the next three years [89].

One of the more robust investigations evaluating this concept followed over 13,000 subjects over 50 years, investigating the relationship between late-life anxiety, depression, and the occurrence of AD [90]. It was concluded that those experiencing late-life depression had a two-fold elevated risk of developing dementia, holding true with previous investigations [89,91,92]. However, this effect was isolated only to late-life depression; having depression early in life was not predictive of future ADRD issues, while having depression during midlife increased the risk of ADRD diagnosis by approximately twenty percent. Nevertheless, when looking specifically at vascular dementia, those in midlife and late-life exhibiting depression symptoms had more than a 3-fold increase in disease risk [90]. The Alzheimer’s Disease Neuroimaging Initiative has also provided a great opportunity for researchers to explore the relationship between mental health and cognitive decline [93]. Initial work by Lee et al. [94] found that subjects suffering from depression experienced greater levels of white matter atrophy in the frontal, parietal, and temporal lobes when compared to individuals without depressive symptoms.

Given the effects that stress, depression, and anxiety appear to have on future risk of cognitive decline, a few investigations have emerged evaluating nutritional interventions, primarily focused on the amino acid tryptophan. In vivo, tryptophan acts as a precursor to serotonin [95] which is synthesized in the central nervous system and the gastrointestinal tract, while peripherally produced serotonin cannot cross the blood–brain barrier [96]. Additionally, tryptophan cannot be synthesized de novo and as a result, adequate levels must be obtained from the diet [97]. Low tryptophan intake leads to lower brain serotonin levels, which is believed to be an important risk factor involved in the development of depression and anxiety [21].

When evaluating data from the NHANES 2001–2012 (*n* = 29,687), it was determined that tryptophan intake was inversely associated with self-reported level of depression [22]. This relationship was also present in an investigation evaluating almost 8000 women, where depressed subjects consumed significantly lower levels of tryptophan than non-depressed subjects [23]. Furthermore, a study from the University of North Dakota evaluated the effects of dietary tryptophan on affective disorders (anxiety, depression, and mood). After consuming a high tryptophan and a low tryptophan diet each for four days (a 2-week washout was included between conditions), the participants’ moods significantly increased with simultaneous decreases in depression and anxiety symptoms [21].

Another amino acid with the potential to modulate symptoms of depression, anxiety, and stress is GABA. While not considered a traditional amino acid, as it cannot be used as a building block for proteins, GABA acts as the major inhibitory neurotransmitter in the brain and decreased levels are implicated in a wide range of issues (e.g., depression and anxiety) [98,99]. This concept was further solidified through a 2010 study suggesting that people suffering from major depression were more likely to have low GABA levels [100], providing further support for the concept. Regardless of the link between GABA levels and mental health symptoms, there is a lack of research on the efficacy of oral supplementation to alleviate such issues. Until future research can determine the effectiveness of exogenous supplementation/intake, it is not recommended to utilize increased intake of GABA as a method of depression and/or anxiety mitigation.

## 6. Protein Intake, Cognitive Health, and the Microbiome

A final comment should address the burgeoning field of the microbiome as it pertains to ADRD. The microbiome includes microorganisms (i.e., bacteria, fungi, viruses, etc.) which may present as helpful or harmful depending on specific volumes and ratios [101]. The human body contains more than 10× the number of microbial cells than human cells and recent research indicates that the gut microbiota can affect the production of chemicals in the brain, potentially contributing to neurological issues such as ADRD [102,103,104].

Regarding ADRD, it appears that there is a back and forth communication system between the microbiome and brain and under disease conditions this communication becomes disrupted, reinforcing harmful pathology promoting pathways [103]. Abnormal activity in the brain may shift gut conditions, leading to a rise in neuroinflammation and amyloid deposits [102]; subsequently, this change in the microbiome increases the production of metabolites in the brain that exacerbates neuroinflammation, anxiety, and depression [102,104]. As a result, it is not surprising that brain health is intricately linked to an individual’s holistic health status.

As with the aforementioned risk factors associated with the development of ADRD, the microbiome is intimately affected by nutrition and specifically protein consumption [105,106,107]. As it pertains to amino acids, the source, concentration, and balance of dietary intake are primary factors contributing to the composition, structure and function of the microbiome. It has been suggested that a suitable ratio between protein and carbohydrate (or even a low protein diet) is recommended as compared to a diet in excess of protein recommendations/requirements [101,107]. In fact, greater dietary protein intake may lead to an increase in pathogenic microorganisms associated with higher risk of disease [101]. However, it is important to note that this mechanism is not well understood and some proteins may have positive effects on microbiome health [107]. For example, bacterial fermentation in the gut of indole (an essential amino acid derived from tryptophan) may improve neural development, protecting against conditions such as multiple sclerosis [101]. As a result of these data, it is important that the mechanistic relationship between protein and the microbiome and its effects on ADRD are further examined before drawing strong conclusions.

## 7. Protein/Amino Acid Intake Recommendations for Older Adults

There is an ongoing debate about the consumption of protein within the human body as it pertains to muscle growth. One argument suggests that there is an upper limit of protein that can be consumed and utilized within a specified timeframe [108], and the other suggests that protein intake is linearly related to protein synthesis and there is no upper threshold in which further intake is no longer effective [109]. Regardless, there is overwhelming evidence that older adults who consume more protein have a greater ability to maintain muscle mass and strength [110,111,112,113]. The European Society for Clinical Nutrition and Metabolism (ESPEN) recommends that aging adults maintain a diet including at least 1.0–1.2 g protein/kg body weight/day [19]. For older adults who may have acute or chronic illnesses, 1.2–1.5 g protein/kg body weight/day is recommended, with even higher intake for individuals with severe illness or injury. However, it should be noted that there are currently no recommendations for protein intake with specific regard to cognition.

Due to the dearth of evidence directly linking consumption of protein/amino acids and cognition (all current research focuses on disease-associated risk factors), it is not yet possible to provide specific recommendations for cognitive health. Perhaps a proper parallel for future guidelines can be found in the current recommendations utilized to help avoid preoperative malnutrition. Global prospective cohort studies have suggested that malnourished hospitalized and surgical patients exhibit significantly worse clinical outcomes such as greater complication risk [114,115,116,117], increased re-admission rates [114,118,119,120], prolonged hospital stays [114,115,118,119,121], increased mortality risk [114,119,122,123], and cost of care [114,124]. As a result, surgical nutrition guidelines, such as the American Society of Parenteral Enteral Nutrition [125], American Society for Enhanced Recovery with Perioperative Quality Initiative [126], and the aforementioned ESPEN [127] have all provided details about the selection of screening tools for nutritional status, tools to assess malnutrition, and proper interventions for at-risk individuals. These guidelines suggest systematic, routine nutritional screenings, subsequent nutrition assessment via validated tools, or a comprehensive nutrition assessment by a registered dietician (when screenings identify risk). As it pertains to the field of cognitive decline, this framework provides an ideal opportunity to lead the development of nutritional guidelines and intervention recommendations for those at risk for ADRD.

## 8. Conclusions

As reviewed above, a large research base has demonstrated that the causes of ADRD are multifactorial and a combination of risk factors (modifiable and unmodifiable) dictate disease onset and progression [11]. Dietary protein and its constituent amino acids may play an important role in long-term cognition through the effects they have on risk factors associated with cognitive decline. To date, there are limited scientific data directly linking protein/amino acid intake to cognitive decline. As a result, most of the conclusions that can currently be made are cursory and require further investigation. For example, it is known that protein can help maximize physical activity results and physical activity is beneficial for maintaining cognitive status; but a direct mechanism of action between protein and cognitive status remains unclear. This concept of elucidating the true mechanisms behind amino acid/protein intake and cognitive health holds true for other modifiable risk factors such as sleep, stress, and mental health. Further research is required directly investigating the downstream results that protein and its constituent amino acids may have on longitudinal cognitive health. Additionally, there is sparse information regarding optimum protein intake requirements as it specifically relates to cognitive health. As a result, additional research is needed to develop definitive conclusions and specific recommendations regarding protein intake or intake of specific amino acids for maintaining optimal cognitive functioning.

## Figures and Tables

**Table 1:** Reasons for diminished protein intake in older adults.

Reduction in oral health making it harder to chew meat-based foods
Mitigated feelings of hunger leading to decreased overall intake
Reduced sense of taste resulting in greater sugar/processed food intake
Reduced sense of smell leading to a decreased enjoyment of certain foods
Reduced ability to shop independently resulting in reduced ability to acquire certain foods
Limited comfort to handle food preparation leading to consumption of more pre-prepared food options

**Table 2:** The relationship between sleep duration and dietary intake: study design recommendations for future investigations *.

**Study Design**
Objectively assess sleep variables (including duration)
Include simultaneous collection sleep/diet data
Include daytime sleep as well as nighttime sleep
Account for relevant genetic variants
Conduct sleep extension trials as well as intervention studies
Conduct longitudinal investigations assessing changes in sleep duration on dietary intake
Objectively measure dietary intake, accounting for timing, consumption frequency, and snacking
**Data Analysis**
Use established cutoffs to define short sleep duration
Test for nonlinear associations between sleep and other outcome variables
Account for seasonality when it comes to assessment timing and collection of data
Test for effect modifiers (i.e., age, sex, BMI, race/ethnicity, or disinhibition)
Account for confounding variables when conducting multivariate analyses
Use findings from small-scale investigations to develop larger cohort studies in order to test causality/mechanisms

* Table is adapted from the original work by Dashti et al. [18].
